# Comorbid pain and falls among Chinese older adults: the association, healthcare utilization and the role of subjective and objective physical functioning

**DOI:** 10.1186/s12877-023-03901-6

**Published:** 2023-05-12

**Authors:** Haocen Wang, Rumei Yang, Yang Yang, Yao Meng, Sha Li, Yun Jiang

**Affiliations:** 1 School of Nursing, Purdue University, Indiana, USA; 2 School of Nursing, Nanjing Medical University, 101 Longmian Avenue, Jiangning District, Jiangsu, Nanjing, 211166 China; 3 School of Nursing, University of Michigan, Michigan, USA

**Keywords:** Pain, Falls, Subjective measure, Objective measure, Physical function

## Abstract

**Background:**

Pain and falls are significant disabling health conditions which cause substantial economic burdens on older adults and their families. Physical functioning (both subjective and objective) might play a significant role in older adults’ pain and falls. In this study we aimed to examine: (1) the relationship between pain and falls among Chinese older adults; (2) pain-fall status (i.e., comorbid pain-fall, pain-only, fall-only, and neither-pain-nor-fall) in relation to healthcare utilization; and (3) whether physical functioning measured either subjectively or objectively would contribute differently to the pain intensity and to the occurrence of falls.

**Methods:**

We used a nationally-representative sample of older adults from the 2011–2012 baseline survey of the China Health and Retirement Longitudinal Study (N = 4,461, aged 60–95 years). Logistic, linear, and negative binomial models adjusted for demographic variables were used in the analysis.

**Results:**

Overall, 36% of older adults reported pain, 20% had fall occurrences, and 11% had comorbid pain and falls. Pain intensity was significantly associated with falls. Individuals in groups of pain-only, fall-only, and comorbid pain-fall reported significantly higher healthcare utilization, that is, more frequent inpatient care and doctor visits than those in the neither-pain-nor-fall group. Subjective, not objective, physical functioning was associated with pain and falls.

**Conclusion:**

Pain and falls are significantly associated with each other, and both can lead to increased healthcare utilization. Compared to objective physical functioning, subjective physical functioning is more likely to correlate with pain and falls, suggesting that self-reported physical status should be considered when designing pain-fall preventive strategies.

**Supplementary Information:**

The online version contains supplementary material available at 10.1186/s12877-023-03901-6.

## Introduction

Pain, a known risk factor for disability [1], impacts 50% of community-dwelling older adults and 70% of long-term care residents [2]. Fall, another significant contributor to disability, affects 30% of community-dwelling older adults and 75% of long-term care residents [3]. The prevention and management of pervasive pain and falls among older adults contribute significantly to high-cost healthcare utilization, which is an issue of primary interest to health systems and policymakers as has been heavily discussed in the literature [4–9]. In contrast to accumulating evidence of the associations of pain and falls with disability [1, 10–13], until recently less research has focused on whether pain is associated with the occurrence of falls among older adults. Most of studies found that older adults with pain had a significant higher risk for falls than those without pain [14–18]. However, a recent study conducted by Ogliari et al. shows that mild pain has no association with fall risk [19]. It is important to consider the pain intensity when studying the relationships between pain and falls. Moreover, among those studies that assessed pain-fall associations, few have explored pain-fall status in relation to healthcare utilization. It remains unclear whether comorbid pain and falls are related to high-cost healthcare service utilization, warranting further investigation.

Pain may be associated with the occurrence of falls, but how exactly they are associated is still unclear. It is commonly observed that they are both associated with functional impairment and disability among older adults [1, 10–13]. When considering functional impairment with pain, it is worth noticing there are two different ways to measure physical functioning in older adults [5]. The subjective measure relies on self-report (e.g., mobility questionnaire), which is widely used but often criticized for recall bias and social desirability factors. The objective measure is performance-based (e.g., walk speed), which is limited to participants’ willingness and capacity to complete those tests. The discrepancies between subjective and objective measures of physical functioning are common in older adults [20–22], making it challenging to determine assessment accuracy. Physical functioning with pain can further complicate the measurement of physical functioning since pain is inherently subjective and has complex mechanisms; pain can also affect an individual’s perceptions of pain-related outcomes [1, 5, 23, 24], possibly including falls. It is also challenging to determine physical function if falls co-occurred to those with functional impairment given that falls are complex and involve multiple risk factors (e.g., physical, psychological, social, and economic components) that can affect an individual’s perceptions of their health (e.g., pain). Thus, an important question remains unanswered, that is, whether the way functional impairment is measured plays a significant role in interpreting its relationships with health outcomes (e.g., pain and falls). In other words, whether different measures of physical functioning would contribute differently to falls among older adults with pain, or to pain among older adults with falls.

To address all these questions, we used a nationally-representative sample of Chinese older adults to (1) explore the relationship between pain intensity and the occurrence of falls among Chinese older adults; (2) describe pain-fall status (e.g., comorbid pain-fall, pain-only, fall-only, and neither-pain-nor-fall) in relation to healthcare utilization; and (3) investigate whether physical functioning measured either subjectively or objectively would contribute differently to pain (specifically pain intensity) among those with falls and to the occurrence of falls among those with pain. Prior evidence reported that falls often occur in those with lower extremity functional impairment [25, 26]. Considering different physical functioning at upper versus lower extremity, we further categorized functional status into upper and lower extremity functioning for both self-reported and performance-based measures. We hypothesized that older adults with pain would have an increased risk for falls. Compared to the neither pain nor falls group, individuals with pain alone, falls alone, or comorbid pain and falls would have increased healthcare utilization measured by total hospitalization costs, the number of times receiving inpatient care, and doctor visits. We also hypothesized that both subjectively and objectively measured physical functioning would be associated with pain intensity among Chinese older adults with falls, and with the occurrence of falls among those older adults with pain.

## Methods

### Data sources

In this population-based study, we used data from the 2011–2012 baseline survey of the China Health and Retirement Longitudinal Study (CHARLS), which is a prospective longitudinal study involving community-dwelling adults aged 45 years and older. CHARLS was designed in collaboration with the Health and Retirement Study (HRS) in the United States in order to develop a robust scientific database to understand the consequences of global aging and also to facilitate the comparability of data across different countries [27]. For this study, we only used respondents aged 60 years and older which gave us a total of 4,461 respondents in the final analysis. A detailed description of the CHARLS was published previously [27].

### Measurements

*Falls.* In CHARLS, participants were asked to answer, “Have you fallen down in the last two years?” with the responses being dichotomized into 1 = yes vs. 0 = no.

*Pain.* Pain was measured using two questions, the first question asking if participants were “often troubled with body pain” (1 = yes, 0 = no) and the second one concerning “how bad the pain is (1 = mild, 2 = moderate, and 3 = severe)” if they answered yes to the first question. We combined these two questions into one measure to represent pain intensity (0 = no pain, 1 = mild pain, 2 = moderate pain, and 3 = severe pain), with higher scores indicating higher levels of pain.

*Comorbid pain and falls*. To better describe pain-fall comorbidity profiles in Chinese older adults, we created a new variable to indicate subgroup status, including 0 = neither-pain-nor-fall group, 1 = comorbid pain-falls group, 2 = pain-only group, and 3 = fall-only group.

*All-cause healthcare utilization.* All-cause healthcare utilization was measured by the following categories: the number of times that participants received inpatient care in the last year; the number of doctor visits in the last month at facilities such as general hospitals, specialized hospitals, community healthcare centers, township hospitals, health care posts, village clinics, and any other type of healthcare facility; and total hospitalization costs in the last year.

*Physical functioning*. Physical functioning is measured by self-reported questionnaires and performance-based tests. Self-reported functional status consisted of (1) upper-extremity functioning, which was measured by a 3-item summary score of the participant’s reports on any difficulty with upper-body mobility activities, including lifting 10 jin, extending arms up, and picking up a coin, with higher scores denoting greater functional difficulties (Cronbach’s alpha = 0.57); and (2) lower-extremity functioning, which was measured by a 4-item summary score of the participant’s reports on any difficulty with lower-body mobility activities, including walking 100 m, climbing several flights of stairs without resting, getting up from a chair after sitting for a long period, and stooping, kneeling or crouching, with a higher score indicating greater functional difficulties (Cronbach’s alpha = 0.75). A comparable measure of performance-based functional status encompassed the tests of grip strength and walking speed. Grip strength was tested twice for each hand at the standing position using a hand-held dynamometer (Yuejian™ WL-1000, Nantong, China). Consistent with prior studies [28, 29], the average value of four tests was used to indicate upper-extremity functioning, with a higher score indicating better physical performance [30]. Walking speed, a simple and powerful measure of the overall quality of gait and motor function [31], was assessed by a 2.5-m walk test that asked participants to walk round twice (there and back) at their usual pace. Consistent with prior research [32], the mean of the two tests was used in the analysis, with a higher score indicating poorer physical performance. All the performance-based tests were performed by trained staff in CHARLS.

*Covariates.* Covariates included *age* (years), gender (0 = male, 1 = female); *educational background* (0 = less than upper secondary, 1 = upper secondary or higher); *marriage* (0 = separated, divorced, widowed, or never married, 1 = married or partnered); *annual income* (0 if annual income ≤ ¥1000, 1 if annual income ≤ ¥6000, and 2 if annual income > ¥6000); *rural residency* (0 = rural residency, 1 = urban residency); *health insurance* (1 if participants were covered by public health insurance, otherwise = 0); *depressive symptoms* measured using the 10-item Center for Epidemiological Studies Depression Scale (CESD-10) [33, 34], and *comorbidity status* measured by the sum score of the number of positive responses to the question, “Have you been diagnosed with … by a doctor?” for 13 common chronic conditions including hypertension, diabetes, cancer, chronic lung disease, cardiac disease, stroke, psychiatric problems, arthritis, dyslipidemia, liver disease, kidney disease, stomach or digestive disease, and asthma.

### Statistical analysis

Preliminary analyses on sociodemographic characteristics of the entire sample, and by pain-fall status, were performed using χ^2^ test and one-way ANOVA as appropriate to compare the outcomes between different pain-falls groups. To test the hypothesis on the association between pain and falls, logistic regression analysis was performed, controlling for demographic variables (e.g., age, gender, educational background, marital status, annual income, rural residency, health insurance, and comorbidity status). To compare the healthcare utilization among different pain-falls groups, linear regression models were fit using the total hospitalization costs as the outcome, negative binomial models with incidence rate ratios (IRR) were fit using the number of times that participants received inpatient care in the last year as the outcome, and the number of times that participants visited a doctor in the last month as the outcome. Negative binomial models for count variable were used in the analysis to account for the violation of Poisson assumption that mean must be equal to its variance. Finally, to understand the relative contributions of objective and subjective domains of lower-extremity and upper-extremity functioning to the occurrence of falls among older adults with pain, logistic or linear regression as appropriate was performed in model 1, followed by model 2 that controlled for demographic variables (e.g., age, gender, education, marriage). Similarly, to understand the relative contributions of objective and subjective domains of lower-extremity and upper-extremity functioning to pain intensity among older adults with falls, linear regression model was performed in model 1, followed by model 2 that controlled for demographic variables (e.g., age, gender, education, marriage). We also conducted a sensitivity analysis to understand the different contributions of subjective and objective lower-extremity and upper-extremity functioning to comorbid pain and falls on the entire sample by creating a new outcome variable based on pain-fall status (1 = having comorbid pain and falls, 0 = not having comorbid pain and falls). The results were consistent with that of the pain or fall sample (see Appendix Table A). Since the missing percentage is less than 8% for each variable of interest (see Appendix Table B), we handled the missing data by pairwise deletion. All statistical analyses and data screening were performed using the R Statistics program with lm, glm, and glm.nb function (R Core Team 2015). The significance level was *P* < 0.05 (two-tailed).

### Ethical consideration

The Biomedical Ethics Review Committee of Peking University approved the CHARLS (the ethical approval number: IRB00001052-11015). All participants provided written informed consent. The use of the de-identified data for this study was reviewed and exempted by the lead author’s University Ethics Committee.

## Results

### Sample description

Table 1 displays descriptive statistics for the entire sample (N = 4,461) and four sub-groups stratified by pain-fall status, including neither-pain-nor-fall-group (n = 2,382, 53.4%), comorbid pain-falls group (n = 468, 10.5%), pain-only group (n = 1,125, 25.2%) and fall-only group (n = 417, 9.3%). Overall, approximately 36% (n = 1,619) of older adults reported pain, and 20% (n = 887) had the occurrence of falls in the last two years. The mean age of the entire sample was 67.84 years (SD = 6.45) with equal distribution between men and women (50%); most were married or partnered (78.9%), lived in rural areas (66.3%), received less than ¥1000 annual income (93.2%), and were covered by public health insurance (93.2%). There were group differences in almost all demographic factors, except for marital status and health insurance. In general, participants with neither pain nor falls were younger, less likely to be female, and more likely to have higher levels of education than their peers in all the other groups. Compared with the group with no pain or falls, all the groups with pain, the occurrence of falls, or both had greater depressive symptoms, comorbidities, functional difficulties (except for walking speed), and greater healthcare utilization. One of the most dramatic differences between the four groups was that participants in the fall-only group reported greater total healthcare costs than those in the co-morbid group and pain alone group.

**Table 1:** Descriptive statistics of Chinese older adults aged ≥ 60 Years (N = 4,461)

	Overall		Stratified by pain-fall status				
	(N = 4,461)		Neither-pain-nor-fall	Comorbid pain-fall	Pain-only	Fall-only	*p*
			(n = 2,382)	(n = 468)	(n = 1,125)	(n = 417)	
Age, mean (SD)	67.84(6.45)		67.69(6.40)	68.14(6.63)	67.41(6.26)	68.97(6.54)	< 0.001
Female, n (%)	2231(50.0)		1047.00(44.00)	309.00(66.00)	622.00(55.30)	212.00(50.80)	< 0.001
Married, n (%)	3518(78.9)		1903.00(79.90)	358.00(76.50)	894.00(79.50)	329.00(78.90)	0.422
Upper secondary or higher education, n (%)	211(4.7)		157.00(6.60)	11.00(2.40)	22.00(2.00)	21.00(5.00)	< 0.001
Rural residency, n (%)	2958(66.3)		1474.00(61.90)	355.00(75.90)	821.00(73.00)	255.00(61.20)	< 0.001
Had public insurance, n (%)	4140(93.2)		2204.00(92.90)	442.00(94.60)	1042.00(93.00)	390.00(93.80)	0.528
Annual income, n (%)							< 0.001
≤ ¥1000	4156(93.2)		2196.00(92.20)	448.00(95.90)	1064.00(94.60)	383.00(91.80)	
≤ ¥6000	124(2.8)		65.00(2.70)	10.00(2.10)	36.00(3.20)	12.00(2.90)	
> ¥6000	178(4.0)		120.00(5.00)	9.00(1.90)	25.00(2.20)	22.00(5.30)	
Had pain, n (%)	1619 (36.3)		-	-	-	-	
Had falls, n (%)	887 (19.9)		-	-	-	-	
Comorbidities, mean (SD)	1.71(1.47)		1.39(1.32)	2.30(1.58)	2.13(1.52)	1.71(1.52)	< 0.001
CESD-10, mean (SD)	9.23(6.49)		6.96(5.46)	13.90(6.43)	12.12(6.46)	9.19(6.13)	< 0.001
Healthcare utilization, mean (SD)							
# of hospitalization	0.17(0.56)		0.11(0.41)	0.25(0.63)	0.23(0.76)	0.20(0.56)	< 0.001
# of doctor visits	0.51(1.56)		0.31 (1.03)	0.78 (2.02)	0.79 (1.93)	0.60 (2.13)	< 0.001
Total hospitalization costs	819.57(4978.67)		678.03(5024.52)	881.99(3235.63)	876.12(4021.17)	1428.07(8036.34)	0.043
Objective physical status, mean (SD)							
Grip strength	26.37 (9.49)		27.96(9.51)	23.07(8.84)	24.93(9.06)	25.38(9.28)	< 0.001
Walking speed	4.80 (7.14)		4.72(9.46)	5.06(2.60)	4.88(2.53)	4.67(2.35)	0.786
Subjective physical status, mean (SD)							
Upper-extremity function	0.37 (0.71)		0.21(0.57)	0.72(0.89)	0.51(0.79)	0.41(0.73)	< 0.001
Lower-extremity function	1.26 (1.21)		0.83(1.05)	2.08(1.11)	1.77(1.17)	1.27(1.19)	< 0.001

**Note.** Coding of variables: gender (0 = male, 1 = female); educational background (0 = less than upper secondary, 1 = upper secondary or higher education); marriage (0 = separated, divorced, widowed, or never married, 1 = married or partnered); annual income (0 = annual income ≤¥1000, 1 = annual income ≤¥6000, 2 = annual income >¥6000); rural residency (0 = rural residency, 1 = urban residency); health insurance (1 = participants was covered by public health insurance, 0 = participants was not covered by public health insurance). CESD-10 = the 10-item Center for Epidemiological Studies Depression Scale

### Associations between pain intensity and falls

Table 2 shows that pain intensity was significantly associated with the occurrence of falls among Chinese older adults (OR = 1.28, 95% CI = 1.20, 1.37, p < 0.001) after controlling for demographic factors including age, gender, education, rural residency, annual income, health insurance, comorbidity, and depressive symptoms. Specifically, older adults with a higher pain intensity level were 1.28 times more likely to suffer from falls.

**Table 2:** Associations between pain intensity and falls among Chinese older adults (N = 4,461)

Variables	OR	95% CI	*p*
Pain intensity	1.28	1.20, 1.37	< 0.001
Age (years)	1.03	1.02, 1.04	< 0.001
Female	1.41	1.19, 1.66	< 0.001
Married/partnered	1.12	0.91, 1.37	0.280
Upper secondary or higher education	1.05	0.69, 1.55	0.819
Rural residency	1.01	0.85, 1.20	0.931
Had public insurance	1.28	0.93, 1.79	0.148
Annual income			
≤ ¥1000	ref		
≤ ¥6000	1.13	0.68, 1.81	0.624
> ¥6000	1.33	0.86, 2.01	0.187
Comorbidities	1.09	1.03, 1.15	0.002
CESD-10	1.04	1.03, 1.06	< 0.001

**Note**. CESD-10 = the 10-item Center for Epidemiological Studies Depression Scale; OR = odds ratio

### Associations between pain-fall status and healthcare utilization

Table 3 describes the association between pain-fall status and healthcare utilization among Chinese older adults. Specifically, compared with the group with neither pain nor falls, groups with pain, falls, and both reported receiving inpatient care a higher number of times in the last year (IRR = 1.55, 95% CI = 1.22–1.97, p < 0.001; IRR = 1.60, 95% CI = 1.16–2.21, p < 0.001; IRR = 1.55, 95% CI = 1.13, 2.13, p = 0.010, respectively), and higher numbers of doctor visits in the last month (IRR = 2.09, 95% CI = 1.71–2.57, p < 0.001; IRR = 1.93, 95% CI = 1.45–2.55, p < 0.001; IRR = 1.82, 95% CI = 1.37–2.41, p < 0.001, respectively). With respect to the total hospitalization costs, only those in the fall-only group reported higher costs than those in the neither pain nor fall group (β = 761.71, 95% CI = 207.82-1315.61, p = 0.007), but the two other groups (comorbid pain & falls group and pain only group) did not report significantly higher costs than the neither pain nor fall group (β = 119.18, 95% CI=-281.77-520.13, p = 0.560 for pain-only group; β = 132.07, 95% CI=-426.44, 690.58, p = 0.643 for comorbid group, respectively).

**Table 3:** Association between pain-fall status and healthcare utilization among Chinese older adults (N = 4,461)

Variables	Total hospitalization costs (n = 4,159)				Number of inpatient cares (n = 4,192)				Number of doctor visits (n = 4,137)		
	β	95% CI	*p*		IRR	95% CI	*p*		IRR	95% CI	*p*
Pain-fall status											
Neither pain nor falls	ref				ref				ref		
Comorbid pain &falls	132.07	-426.44, 690.58	0.643		1.55	1.13, 2.13	0.010		1.82	1.37, 2.41	< 0.001
Pain-only	119.18	-281.77, 520.13	0.560		1.55	1.22, 1.97	< 0.001		2.09	1.71, 2.57	< 0.001
Fall-only	761.71	207.82, 1315.61	0.007		1.60	1.16, 2.21	< 0.001		1.93	1.45, 2.55	< 0.001
Age, years	15.17	-10.25, 40.59	0.242		1.02	1.01, 1.04	< 0.001		1.00	0.98, 1.01	0.550
Female	-386.18	-710.04, -62.32	0.019		0.80	0.65, 0.97	0.030		0.98	0.82, 1.16	0.780
Married	334.81	-73.03, 742.65	0.108		1.42	1.09, 1.84	0.010		0.98	0.79, 1.21	0.820
Upper secondary or higher education	1035.05	301.59, 1768.50	0.006		1.41	0.92, 2.14	0.110		0.84	0.56, 1.28	0.420
Rural residency	-361.35	-699.09, -23.61	0.036		0.92	0.75, 1.13	0.430		0.92	0.77, 1.10	0.350
Had public insurance	529.96	-87.33, 1147.26	0.092		1.50	0.97, 2.31	0.070		1.42	1.01, 2.00	0.050
Annual income											
≤ ¥1000	ref				ref				ref		
≤ ¥6000	-187.49	-1114.10, 739.12	0.692		0.66	0.33, 1.31	0.230		0.89	0.54, 1.47	0.650
> ¥6000	-485.88	-1284.52, 312.76	0.233		0.55	0.3, 1.01	0.050		0.68	0.43, 1.08	0.110
Comorbidities	272.46	161.38, 383.54	< 0.001		1.34	1.26, 1.43	< 0.001		1.19	1.12, 1.26	< 0.001
CESD-10	1.31	-25.77, 28.39	0.925		1.03	1.01, 1.04	< 0.001		1.02	1.01, 1.04	< 0.001

**Note.** β = Unstandardized coefficient; IRR = Incidence rate ratios; CI = confidence interval; CESD-10 = the 10-item Center for Epidemiological Studies Depression Scale

### The relative contributions of subjective and objective physical functioning

Table 4 shows the results of the relative contributions of subjective and objective physical functioning (including upper-extremity and lower-extremity functioning) to falls among a sub-sample of Chinese older adults with pain. Model 1 suggested that self-reported upper-extremity (OR = 1.30, 95% CI = 1.10–1.52, p = 0.002) and lower-extremity functional status (OR = 1.20, 95% CI = 1.07–1.34, p = 0.001) were statistically associated with falls, and that performance-based grip strength (OR = 0.98, 95% CI = 0.97-1.00, p = 0.026) was marginally associated with falls but not the walking speed (OR = 0.98, 95% CI = 0.94–1.03, p = 0.516). However, these associations only persist in self-reported measures of functioning, including upper-extremity (OR = 1.31, 95% CI = 1.11–1.55, p = 0.002) and lower-extremity functioning (OR = 1.15, 95% CI = 1.02–1.30, p = 0.020) after adjustment for demographic covariates (e.g., age, gender, education, and marital status). Similar findings were seen regarding the contributions of functioning to pain in those older adults with falls, that is, subjective physical functioning including upper (β = 0.17, 95% CI = 0.05–0.28, p = 0.005) and lower-extremity (β = 0.19, 95% CI = 0.11–0.26, p < 0.001) were statistically associated with pain among those with falls, but no statistical association of grip strength (β = 0.00, 95% CI=-0.01-0.01, p = 0.998) and walking speed (β=-0.01, 95% CI=-0.05-0.02, p = 0.439) with pain (see Model 2, Table 5).

**Table 4:** The relative contributions of subjective and objective physical functioning to falls among Chinese older adults with pain (n = 1,619)

Variables	Model 1				Model 2		
	OR	95%CI	*p*		OR	95%CI	*p*
Grip strength	0.98	0.97,1.00	0.026		0.99	0.98, 1.01	0.496
Walking speed	0.98	0.94,1.03	0.516		0.97	0.92, 1.02	0.242
Upper-extremity function	1.30	1.10,1.52	0.002		1.31	1.11, 1.55	0.002
Lower-extremity function	1.20	1.07,1.34	0.001		1.15	1.02, 1.30	0.020
Age (years)					1.02	1.001, 1.04	0.037
Female					1.45	1.07, 1.98	0.017
Married					1.02	0.75, 1.39	0.922
Upper secondary or higher education					1.27	0.48, 3.03	0.611
Rural residency					1.26	0.95, 1.68	0.119
Had public insurance					1.38	0.83, 2.39	0.235
Annual income							
≤ ¥1000					ref		
≤ ¥6000					0.99	0.43, 2.07	0.988
> ¥6000					1.21	0.49, 2.71	0.654
Comorbidities					1.04	0.96, 1.13	0.348
CESD-10					1.02	1.003, 1.04	0.020

**Note.** CESD-10 = the 10-item Center for Epidemiological Studies Depression Scale; OR = odds ratio. Sample size for Model 1 and Model 2 were 1,417 and 1,343, respectively

**Table 5:** The relative contributions of subjective and objective physical functioning to pain among Chinese older adults with falls (n = 887)

Variables	Model 1				Model 2		
	β	95%CI	*p*		β	95%CI	*p*
Grip strength	-0.01	-0.02, 0.00	0.150		0.00	-0.01, 0.01	0.998
Walking speed	0.00	-0.04, 0.03	0.875		-0.01	-0.05, 0.02	0.439
Upper-extremity function	0.15	0.03, 0.27	0.014		0.17	0.05, 0.28	0.005
Lower-extremity function	0.29	0.22, 0.37	< 0.001		0.19	0.11, 0.26	< 0.001
Age (years)					-0.01	-0.03, 0.00	0.056
Female					0.17	-0.03, 0.38	0.101
Married					0.05	-0.17, 0.26	0.655
Upper secondary or higher education					0.04	-0.44, 0.51	0.883
Rural residency					0.40	0.22, 0.58	0.000
Had public insurance					-0.03	-0.38, 0.32	0.850
Annual income							
≤ ¥1000					ref		
≤ ¥6000					0.18	-0.34, 0.70	0.491
> ¥6000					-0.36	-0.81, 0.09	0.112
Comorbidities					0.07	0.01, 0.12	0.013
CESD-10					0.04	0.03, 0.06	0.000

**Note.** CESD-10 = the 10-item Center for Epidemiological Studies Depression Scale; β = unstandardized coefficients. Sample size for Model 1 and Model 2 were 791 and 739, respectively

## Discussion

Pain and falls are well-known factors that significantly influence older adults’ physical and mental health, generating substantial economic burden on older adults and their families [1, 3–9]. Physical functioning can be a significant shared factor and play an important role in pain and falls among older adults [1, 10–13]. This study analyzed the baseline data from a nationally-representative survey, the China Health and Retirement Longitudinal Study, and confirmed that both pain and falls are prevalent among Chinese adults aged 60 or older. To our knowledge, this is the first study that directly confirmed the significant associations of pain and falls with each other and with healthcare utilization, and further demonstrated the contributions of objective and subjective physical functioning to pain (specifically pain intensity) and falls among Chinese older adults, given that physical functioning is a shared risk for both pain and falls. Several important findings have emerged from the study.

First, pain and falls are significantly associated with each other. Overall, about 36% of Chinese older adults aged 60 + years reported pain, 20% had the occurrence of falls, and 11% reported the comorbidity of pain and falls in the last two years. Although this analysis did not reveal a high comorbidity rate of pain and falls when compared to that of pain-only and fall-only among Chinese older adults, it demonstrated that as pain intensity increased, older adults were at significantly higher risk for falls, even with demographic factors considered. This finding is similar to what has been found in U.S. older adults [18]. The significant positive relationship between pain and falls has also been reported in other populations, such as the Malaysian, Mexican, and Australian community-dwelling older adults [35–39]. Due to limited studies on comorbidities of pain and falls, we are not able to directly compare the comorbidity rate in our sample with other samples and to characterize the complex interplays between pain and falls. However, some studies have shown that an exercise-based fall prevention program could decrease older adults’ pain levels as well as reduce fall risk [39, 40]. Our findings together with previous studies suggest that the pain intensity level should also be considered when preventing falls, also raising an important question about how to motivate older adults with pain to exercise to prevent falls. Further studies are needed to develop better pain management to help motivate and facilitate physical activity and thus reduce falls among older adults.

Regarding healthcare utilization, this study confirmed that older adults with pain or/and falls had significantly higher utilization of healthcare services, specifically with more inpatient care and doctor visits, compared to those without any pain or falls. This finding is consistent with those from studies in many other countries [5, 41–43]. With respect to the total hospitalization costs, different from our hypothesis, only the fall-only group had significantly higher hospitalization costs than those without any pain or falls. This study compared hospitalization-related expenditures without including outpatient and self-treatment expenditures. It is possible that, unlike falls that often cause injuries and require immediate medical care (e.g., hospitalization and rehabilitation), older adults with pain primarily use outpatient services and self-treatment rather than hospitalization for pain management [6]. It is also possible that older adults with pain might not seek as much professional help as those with falls. A recent retrospective study in China found that the average hospitalization costs for each older adult patient with fall-related injuries were about 45,000 Chinese Yuan ($7,061) [4]. In contrast, the direct medical cost of pain was about 2628.8–3945.7 Chinese Yuan ($380-$570), and half of the cost was outpatient expenditure [6]. This might explain why the pain-only group reported lower hospitalization costs than the fall-only and comorbid pain-fall groups, although it is not statistically significant. We also speculate that older adults with pain may be more careful with their daily activities to avoid pain [44, 45], and subsequently may reduce the likelihood of getting serious falls. All these findings suggest that the relationship between pain and falls is often complicated. Pain may lead to more falls, but not necessarily more serious falls or higher costs of hospitalization. More studies are required to investigate the complex interplay between pain, the number and the severity of falls, and pain and/or fall-related cost among older adults.

A notable finding in our study is that compared to objectively measured physical functioning, subjectively measured physical functioning uniquely contributed to falls and pain among older adults with pain and falls, respectively. We found that among older adults with pain, only subjectively measured physical functioning, specifically upper-extremity and lower-extremity functioning, was negatively associated with falls. There are no significant relationships between objectively measured physical functioning (i.e., grip strength and walking speed) and falls. Similarly, among old adults who had the occurrence of falls, only subjectively measured physical functioning was negatively correlated to their pain intensity levels. Previous studies found that subjectively and objectively measured physical functioning were only moderately correlated [46] and discrepancies were reported between subjective and objective measures of physical functioning [20–22]. Researchers generally believed that objective measures might be more accurate than subjective measures in capturing an individual’s physical capabilities. Our findings suggest that instead of focusing only on objective indicators of physical functioning, subjective physical functioning should be equally considered. Previous research indicates that a subjective measure of physical functioning is not only based on objective physical functioning but also affected by efficacy beliefs and other factors (e.g., personalities) [20]. Efficacy beliefs as a cognitive appraisal or representation of self-abilities to perform daily activities might influence self-perceived physical functioning, which might not solely be based on objective physical performance [20, 47]. Subjective perception of personal physical functioning can be a more comprehensive biopsychosocial-based measurement that might reflect the fact that both pain and fall are associated with a variety of biopsychosocial factors [41, 48]. Individuals with strong efficacy beliefs often showed better overall adjustment to health conditions [49]. It is not surprising that older adults with strong efficacy beliefs are more likely to take efforts to be physically active despite the pain and the risk for falls. Therefore, including subjective measures as part of older adults’ physical functioning assessment is crucial, particularly among those with comorbid pain and falls.

This study has several limitations. First, the assessment of falls and pain intensity was self-reported by the older adults, which may have introduced recall bias. Second, direct healthcare costs pertaining to pain and falls were not measured in the CHARLS study, which might affect the interpretation of results about overall healthcare utilization. Third, the reliability of self-reported measures of the upper-extremity functioning is relatively low, which might affect the interpretation of results. Fourth, we used the data from a nationally representative survey, which ensured the generalizability of our results. However, since the data was collected during 2011–2012, the validity of our findings should be reassessed by more recent data. Healthcare reforms and healthcare environment changes in China may impact Chinese older adults’ health-related behaviors, especially their healthcare utilization. Finally, because of the use of cross-sectional data in this study, we were not able to identify the causal relationship between pain intensity and falls. Longitudinal studies are needed to further understand pain-fall associations.

## Conclusion

Our findings suggest that pain intensity was significantly associated with falls among Chinese older adults aged 60 years and over. Pain-fall status, including comorbid pain and falls, is associated with increased healthcare utilization, especially with the frequency of inpatient care in the last year and the number of doctor visits in the last month. Further, poor subjectively-measured physical functioning status was associated with falls in those with pain and with higher levels of perceived functional difficulties, denoting a higher likelihood of falls. Likewise, a poor subjective physical functioning status was associated with pain in those with falls and with higher levels of perceived functional difficulties denoting a higher likelihood of pain. Poor subjective physical functioning might be a more important factor than objective physical functioning contributing to both pain and falls among Chinese older adults. Clinicians should carefully assess and improve subjective physical functioning in older adults.

## Electronic supplementary material

Below is the link to the electronic supplementary material.

Appendix Table A. The Relative Contributions of Subjective and Objective Physical Functioning to Comorbid Pain and Falls among Chinese Older Adults (N = 4,461).

Appendix Table B. Missing data of study variables.

## Data Availability

The datasets are publicly available on the CHARLS website https://opendata.pku.edu.cn/dataverse/CHARLS.

## Abbreviations

CESD: Center for Epidemiological Studies Depression Scale
CI: Confidence interval
IRR: Incidence rate ratios
OR: Odds ratio
