# Supplementary material for: Comorbid pain and falls among Chinese older adults: the association, healthcare utilization and the role of subjective and objective physical functioning
Source: BMC Geriatr. 2023 May 12;23:286. doi: 10.1186/s12877-023-03901-6 (PMC10176684; doi:10.1186/s12877-023-03901-6)
Supplement: Supplementary file 1 — Appendix Table A. The Relative Contributions of Subjective and Objective Physical Functioning to Comorbid Pain and Falls among Chinese Older Adults (N = 4,461). [file 12877_2023_3901_MOESM1_ESM.docx]

### Appendix Table A

### The Relative Contributions of Subjective and Objective Physical Functioning to Comorbid Pain and Falls among Chinese Older Adults (N=4,461)

| Variables | Model 1 | | | |  | | Model 2 | | | |
| --- | --- | --- | --- | --- | --- | --- | --- | --- | --- | --- |
|  | OR | 95%CI | *p* |  | | OR | | 95%CI | *p* |  |
| Grip strength | 0.98 | 0.96, 0.99 | 0.000 |  | | 0.99 | | 0.97, 1.01 | 0.243 |  |
| Walking speed | 0.97 | 0.93, 1.00 | 0.200 |  | | 0.97 | | 0.92, 1.01 | 0.153 |  |
| Upper-extremity function | 1.31 | 1.13, 1.50 | 0.000 |  | | 1.28 | | 1.10, 1.48 | 0.002 |  |
| Lower-extremity function | 1.75 | 1.59, 1.94 | 0.000 |  | | 1.49 | | 1.33, 1.66 | 0.000 |  |
| Age (years) |  |  |  |  | | 1.01 | | 0.99, 1.03 | 0.409 |  |
| Female |  |  |  |  | | 1.53 | | 1.16, 2.03 | 0.003 |  |
| Married |  |  |  |  | | 1.08 | | 0.82, 1.45 | 0.578 |  |
| Upper secondary or higher education |  |  |  |  | | 0.86 | | 0.35, 1.80 | 0.718 |  |
| Rural residency |  |  |  |  | | 1.54 | | 1.19, 2.02 | 0.001 |  |
| Had public insurance |  |  |  |  | | 1.34 | | 0.84, 2.25 | 0.246 |  |
| Annual income |  |  |  |  | | 0.99 | | 0.97, 1.01 | 0.243 |  |
| ≤ ¥1000 |  |  |  |  | | ref | |  |  |  |
| ≤ ¥6000 |  |  |  |  | | 1.18 | | 0.53, 2.31 | 0.661 |  |
| > ¥6000 |  |  |  |  | | 1.08 | | 0.47, 2.16 | 0.843 |  |
| Comorbidities |  |  |  |  | | 1.14 | | 1.06, 1.23 | 0.000 |  |
| CESD-10 |  |  |  |  | | 1.07 | | 1.05, 1.09 | 0.000 |  |

**Note.** CESD-10, 10-item Center for Epidemiological Studies Depression Scale; OR, odds ratio. We recoded pain and fall status into a new binary outcome with 1=having comorbid pain and falls, 0=not having comorbid pain and falls.
