# Supplementary material for: Comorbid pain and falls among Chinese older adults: the association, healthcare utilization and the role of subjective and objective physical functioning
Source: BMC Geriatr. 2023 May 12;23:286. doi: 10.1186/s12877-023-03901-6 (PMC10176684; doi:10.1186/s12877-023-03901-6)
Supplement: Supplementary file 2 — Appendix Table B. Missing data of study variables. [file 12877_2023_3901_MOESM2_ESM.docx]

**Appendix Table B**

**Missing data of study variables**

| Variable | Missing | Total | Percent Missing |
| --- | --- | --- | --- |
| Age | 0 | 4,461 | 0 |
| Gender | 0 | 4,461 | 0 |
| Education | 0 | 4,461 | 0 |
| Marital status | 0 | 4,461 | 0 |
| Rural residency | 0 | 4,461 | 0 |
| Had public insurance | 17 | 4,461 | 0.38 |
| Income | 3 | 4,461 | 0.07 |
| Had pain | 14 | 4,461 | 0.31 |
| Had falls | 58 | 4,461 | 1.3 |
| Comorbidities | 183 | 4,461 | 4.1 |
| CESD-10 | 75 | 4,461 | 1.68 |
| # of hospitalization | 5 | 4,461 | 0.11 |
| # of doctor visits | 71 | 4,461 | 1.59 |
| Total hospitalization costs | 43 | 4,461 | 0.96 |
| Grip strength | 176 | 4,461 | 3.95 |
| Walking speed | 324 | 4,461 | 7.26 |
| Upper-extremity function | 1 | 4,461 | 0.02 |
| Lower-extremity function | 1 | 4,461 | 0.02 |

**Note.** CESD-10, 10-item Center for Epidemiological Studies Depression Scale.
